# Understanding Breast Cancer Aggressiveness and Its Implications in Diagnosis and Treatment

**DOI:** 10.3390/jcm12041375

**Published:** 2023-02-09

**Authors:** Nader Afifi, Carlos A. Barrero

**Affiliations:** Department of Pharmaceutical Sciences, Temple University School of Pharmacy, Philadelphia, PA 19140, USA

Breast cancer (BC) is the most common form of cancer in women worldwide. BC aggressiveness is associated with metastasis, relapse, and high mortality rates. The aggressiveness of BC can vary according to the disease stage and other related factors such as glucose metabolism, hormone receptors, and gene signature expression [1]. Clinical characteristics of BC show that it is intrinsically a heterogeneous complex of diseases with specific molecular subtypes. Markers for each subtype are characterized by histological, genomic, and gene expression analysis levels, each of which provides signatures that can be used to categorize breast cancers as luminal A (proximal), luminal B (distal), luminal-HER-2, and basal-like phenotypes [1].

Cancer treatment includes surgery, chemotherapy, radiotherapy, hormone therapy, and immunotherapy. BC treatment is more difficult after metastasis and recurrence [2]. Relapses are linked to multi-drug resistance (MDR) characteristics such as increased efflux transporter expression and activation of transcription factor genes that lead to mutations and proliferation of cancer cells [3]. Current studies to overcome BC MDR include targeted nanomedicine chemotherapy delivery approaches [4].

Triple-negative breast cancer (TNBC) is a highly aggressive phenotype associated with poor prognosis. This condition is characterized by the lack of expression of (1) estrogen receptors (ER), (2) progesterone receptors (PR), and (3) the epidermal growth factor receptor-2 (HER2). The absence of these markers predisposes it to rapid metastasis, treatment resistance, and a high recurrence rate [5]. In addition, the absence of these receptors limits treatment options for TNBC patients. While TNBC can resist hormone- and immunotherapy, it is often sensitive to chemotherapeutic agents and radiotherapy [1]. 

One of the hallmarks of cancer cells is the reprogramming of cellular metabolism in response to the increased energy demand necessary to support proliferation and tumor growth. This process forces cells to depend on glucose as fuel and glycolysis to generate NADPH and ATP instead of the normal oxidative phosphorylation processes, a phenomenon known as the “Warburg effect” [6,7]. Glucose enters cancer cells through specific transporters. The two main routes responsible for glucose transport are the facilitative glucose transporter family (GLUTs) and the sodium-glucose co-transporter family. Since glucose transporters play a key function in BC metabolism, these transporters are considered therapeutic targets for BC aggressiveness [8].

Glycolysis is also induced by oncogenes activation and loss of tumor suppressors, linking a high rate of glucose metabolism and cancer [8]. Since glucose is supporting cancer progression, it has been suggested that controlled calorie intake will reduce oxidative stress and protect cells from becoming oncogenic [9]. Therefore, caloric restriction could ameliorate the risk factors in breast cancer by reducing blood glucose levels, inflammation, and the onset of angiogenesis [10].

Early diagnosis of BC is critical to ensuring a positive patient outcome, and mammograms are the gold standard screening tool for early detection. Unfortunately, only 20% of BC cases are detected at an early stage. Clinical trials designed to assess the utility of potential BC biomarkers, especially in its highly aggressive forms, have helped to shape the treatment management. Tissue plasminogen activator (t-PA) antigen and plasminogen activator inhibitor-1 (PAI-1) have been recently described as biomarkers with high prognostic potential. A clinical study demonstrated that low plasma concentrations of t-PA antigen and higher PAI-1 activity are shown to be strong predictors for distant metastasis and independent prognostic markers in BC patients [2].

Complete tumor molecular profile characterization is a valuable tool for identifying BC markers and potential therapeutic targets. Molecular signature profiling, including transcriptomics, proteomics, and metabolomics, can provide useful tools for tumor classification and support assessing BC aggressiveness and MDR expression. New approaches for novel biomarkers identification using omics technologies enable early BC diagnosis [11]. Identifying specific pathways activated in aggressive BC subtypes can point to novel therapeutic targets and support individualized treatment. Transcriptomics has facilitated theunderstanding of specific BC tumor gene expression related to disease aggressiveness Kinesin Eg5 was found overexpressed in BC and has been proposed as a nobel target for therapeutic inhibition to treat BC patients [12].

Proteomic profiling of plasma samples revealed the significant relationship between the upregulated proteins in breast cancer and their association with the complement system and protease inhibitor activities. Mapping the myriad of proteins and their role in activating or inhibiting the immune system may help address the disease and early detection [13]. Several pathways in the cancer cell or tumor environment are altered in BC [8]. These changes often cause the formation of metabolites in cancer cells that can be distinguished from the metabolites of normal cells [14]. Imaging techniques such as magnetic resonance imaging (MRI) can be used to study increased metabolite uptake by tumor cells, which could translate into earlier diagnosis and improved patient outcomes [15]. Integrated bioinformatics and systems biology can play a role in understanding and testing key expressed genes related to BC recurrence. Therefore, using omics technologies to identify BC specific biomarkers is an indispensable approach for BC early diagnosis that can prevent metastasis, improve treatment, and reduce relapse [13,16].

Despite the efforts and resources invested to improve outcomes for BC patients, significant obstacles remain, some of which could be addressed by identifying pathway profiles and biomarkers for early BC diagnosis. In addition, advances in imaging technologies can aid in drug discovery and development, disease management, and overall patient outcome.
